# Distinct responses of cones and melanopsin-expressing retinal ganglion cells in the human electroretinogram

**DOI:** 10.1186/1880-6805-31-20

**Published:** 2012-06-26

**Authors:** Yumi Fukuda, Shigekazu Higuchi, Akira Yasukouchi, Takeshi Morita

**Affiliations:** 1 Department of Living Environmental Science, Fukuoka Women’s University, 1-1-1, Kasumigaoka, Higashi-ku, Fukuoka 813-8529, Japan; 2 Department of Human Science, Kyushu University, 4-9-1, Shiobaru, Minami-ku, Fukuoka, 815-8540, Japan

**Keywords:** Melanopsin-expressing retinal ganglion cells, Circadian rhythms, Non-visual/visual perception, Electroretinogram

## Abstract

**Background:**

The discovery of the novel photoreceptor, melanopsin-expressing retinal ganglion cells (mRGCs), has raised researchers’ interest in photoreceptive tasks performed by the mRGC, especially in non-image-forming visual functions. In a prior study, we investigated the mRGC response to light stimuli independent of rods and cones with the four-primary illumination system, which modulates stimulus levels to the mRGC and cones independently, and mRGC baseline responses were recorded in the electroretinogram (ERG).

**Methods:**

In the present study, we used the same illumination system to compare independent responses of the mRGC and cones in five subjects (mean ± SD age, 23.0 ± 1.7 years). The ERG waveforms were examined as direct measurements of responses of the mRGCs and cones to stimulation (250 msec). Implicit times (the time taken to peaks) and peak values from 30 stimuli given to each subject were analyzed.

**Results:**

Two distinct positive peaks appeared in the mRGC response, approximately 80 msec after the onset of the stimuli and 30 msec after their offset, while no such peaks appeared in the cone response. The response to the mRGC stimulus was significantly higher than that to the cone stimulus at approximately 80 msec (*P* < 0.05) and tended to be higher than the cone stimulus at approximately 280 msec (*P* = 0.08).

**Conclusions:**

Implicit time of the first peak was much longer than that to the b-wave and this delay might reflect mRGC’s sluggish responses. This is the first report of amplitudes and implicit time in the ERG from the response of the mRGC that is independent of rods and cones, and obtained using the four-primary illumination system.

## Background

Over the last 20 years, researchers have tried to understand the photoreceptor mechanisms which regulate the circadian system. The discovery of the novel photoreceptor, melanopsin-expressing retinal ganglion cells (mRGCs), has raised those researchers’ interest in differences in photoreceptive tasks played by the mRGCs in comparison with rods and cones, especially in non-image-forming visual functions, such as circadian rhythm regulation and the pupillary light reflex [1-4]. Although all of the mechanisms by which the mRGC regulates non-visual/visual functions in humans have not been established, some reports reveal that the mRGCs differ from rods and cones in many respects. For example, they respond to light much more sluggishly [5] and are distributed in the retina much more sparsely [6]. Only a small subset of retinal ganglion cells contains the functional photopigment (melanopsin) and is intrinsically photosensitive [7]. Furthermore, light depolarizes these cells tonically and elevates spike frequency, while the opposite changes occur when rods and cones are stimulated [5,8].

As it is important to understand mRGC characteristics and their role independent of effects due to the rods and cones, mRGC responses should be produced and measured independently of cone and rod responses. In a prior study, we investigated responses to light stimuli with the four-primary illumination system [9,10], which modulates stimulus levels to the mRGC and cones independently, and responses to contrasts, which were stimulus levels of the mRGCs to background, were recorded in the electroretinogram (ERG) [11]. The ERG response to mRGC stimulation rose linearly with the contrast of the stimulus. The purpose of the present study was to quantify ERG responses to stimulating cones and mRGCs independently of one another.

With regard to the ERG response, four major components, the a-, b-, c- and d-waves, are commonly considered [12-14], although their precise origin and meaning remain to be elucidated. An elucidation of how depolarization of mRGCs in response to a light stimulus becomes manifest in the ERG is one of the main interests of the present study. It is possible that the b-wave might be a way to observe mRGC responses in the ERG since, following bright light stimuli, the b-wave implicit time (time taken to reach a peak) in the photopic ERG showed an action spectrum (λmax = 483 nm) [15] and sensitivity that closely matched results for the mRGC spectra from other reports [4,5,8,16,17]. On the other hand, in the fields of visual science and chronobiology, there have been attempts to investigate circadian rhythms in visual function by using the ERG [18-20]. While classical photoreceptors also can be involved in circadian rhythm regulation [17,21], the mRGCs have become a significant factor as the mRGCs have been found to make important contributions to circadian rhythm regulation [1,2]. However, in previous human ERG studies, it was not straightforward to identify the component of the ERG which derived from intrinsic mRGC responses, since ERG responses reflected neural activities of other photoreceptors, rods and/or cones in addition to the mRGCs. In this study, we have used the silent-substitution technique [22] which enables us to control stimulus levels to the mRGC and cones based on calculations of relative luminous efficiency and spectral radiance of the light. In addition, we have used masking-cone stimuli to selectively suppress the cones. It is likely that there are individual differences among subjects in the receptor sensitivity curve due to differences in ocular optical densities. Since the receptor sensitivity curves for a standard observer were used, these individual differences could influence the ERG response. If this were the case, then the mRGC response would be affected by cones, and vice versa. Therefore, selectively suppressing the cone-mediated pathways with a masking-cone paradigm was required.

## Methods

### Apparatus

**Figure 1:**
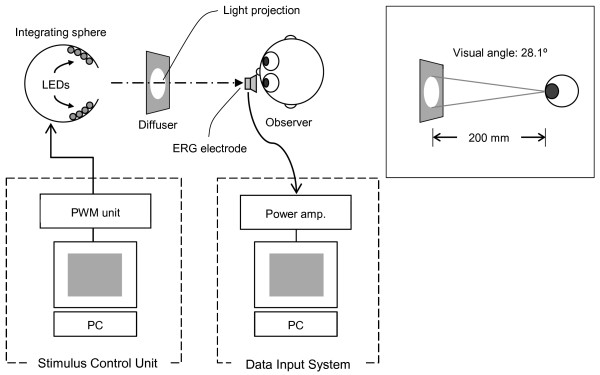
A diagram illustrating the experimental set-up, the integrating sphere exposure and the monitor array.

Figure1 illustrates the apparatus used in the experiment. A personal computer controlled the four-primary illumination system [9]. It consisted of an optical diffuser and an integrating sphere. Four types of light-emitting diode (LED; peak wavelengths: 633 nm, 593 nm, 508 nm and 468 nm; and half-height bandwidth: 13 to 32 nm) were used. The light emitted from four types of LED, which were embedded in the inner wall of the integrating sphere, projected as the internally-synthesized test stimuli. The luminance output of each LED was controlled by both pulse-width modulation units and an embedded controller (H8/3052, Renesas Technology, Tokyo, Japan). A detailed description of the illumination system has previously been published [9,11]. 

### Photoreceptor excitation

Excitation was expressed as stimulus levels to each photoreceptor [11]. The 10-deg cone fundamentals [23,24] and spectral radiance of the light stimuli were used to calculate the excitation of cones sensitive to long (L), middle (M) and short (S) wavelengths. The fundamental, the spectral sensitivity of mRGC, in a 10° field displaying a peak wavelength at 502 nm was used in the present study after careful calibration of prereceptoral filters [9]. Excitation of the mRGC was then calculated from the fundamental (represented as a unity peak) as relative luminous efficiency and the spectral radiance of the light stimuli. The background stimulus had a CIE (Commission Internationale de L’Eclairage) coordinate (CIE 1964) of (0.600, 0.358) and a luminance of 534 cd/m^2^. The receptor excitations for the background were 443 cd/m^2^ for the L cone, 91 cd/m^2^ for the M cone, 30 cd/m^2^ for the S cone and 116 cd/m^2^ for the mRGC.

### Test stimuli

The uniform test stimulus was displayed in a circular region of the diffuser subtending an angle of 28.1° at the eye. Relatively prolonged test stimuli (250 msec) were used in this study, as a brief light flash may induce complex combined responses to the onset and offset of a light stimulus [25,26]. There were two separate test stimuli in the experiment: varied mRGC excitation alone (mRGC stimulus) and varied L-, M- and S-cone excitation alone (cone stimulus). The mRGC stimulus had a Weber contrast of 0.50 for the mRGC, while the contrast for the L, M, and S cones was zero. The cone stimulus had a Weber contrast of 0.30 for luminance, while the mRGC contrast was zero. Since the cone stimulus was designed to stimulate the three types of cones equally, the color of the stimulus was the same as that of the background and kept constant throughout. After we assessed the light stimuli from the integrating sphere with a spectroradiometer (CS-1000A, Konica Minolta, Tokyo, Japan), it was ensured that the contrasts between the exact excitation and theoretical values were within a few percentages.

### Masking-cone stimuli

**Figure 2:**
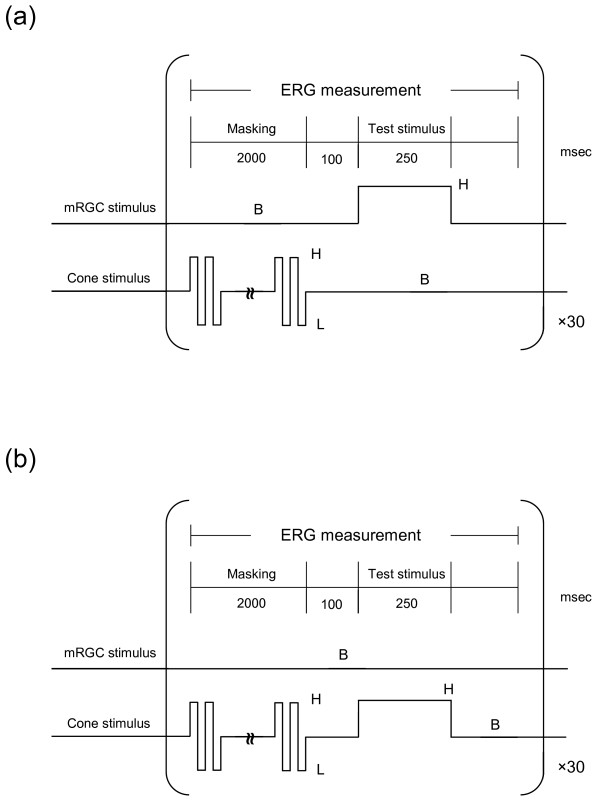
**Protocols for presenting light stimuli (including test stimuli and masking-cone stimuli).** (**a**) An mRGC stimulus with masking stimuli flickering with contrast of ±0.30 modulation at 20 Hz. The stimulus to the mRGC varied from background (B) to high (H) with contrast of +0.50 modulation for 250 msec. (**b**) A cone stimulus with the masking stimuli. The stimulus to the cone varied from background (B) to high (H) with contrast of +0.30 modulation. Each measurement was repeated 30 times.

Figure2 shows the protocol for presenting the light stimuli with masking-cone stimuli. Panel (a) shows an mRGC stimulus with masking-cone stimuli. Panel (b) shows a cone stimulus with the masking stimuli and a constant mRGC stimulus. Each trial consisted of 2,000-msec presentation of the masking stimuli, 100-msec presentation of background (B) stimulus levels, and presentation of a test stimulus for 250 msec. The masking stimuli were presented at 20 Hz as square waves with a Michelson contrast of 0.30. At a high temporal frequency of 20 Hz, sensitivity of the cone is higher than that of the mRGC, as response latency to light is 30 to 40 msec for the cones and 900 msec for those mRGCs which do not receive input from the rods or cones [8]. Therefore, it was expected that the masking-cone stimulus would efficiently stimulate the cone components in the ERG responses. The masking stimulus modulated L, M and S cones with no change in mRGC excitation. This temporally flickering mask can selectively suppress the cone-mediated pathways since it is modulated only in L, M and S cones. 

### Experimental procedure

Neural activity of photoreceptors from five healthy subjects (three males, two females; mean ± SD age, 23.0 ± 1.7 years; Japanese) was analyzed in this study. The subjects were university students who voluntarily joined after we explained the experimental overview and confirmation that all subjects had ocular health and normal color vision according to the Ishihara color blindness test. For test sessions, a mydriatic agent was dropped into the subject’s left eye. After the subjects’ pupils had become completely dilated, the subject entered an artificial climate chamber. Then an ERG electrode (EA-102, Meiyo, Aichi, Japan) was placed in contact with the subject’s left cornea by an ophthalmologist. In the test sessions, the subjects rested their chin on a rest and gazed at a fixation point at the center of circular light stimulus on a diffuser. After five-minute’s adaptation, in order to saturate rod responses and adapt cone responses to a background light stimulus, the test session was started. Four combinations of light stimuli were randomly presented: the mRGC stimulus or the cone stimulus, each with or without the masking-cone stimuli (Table 1). The ERG signals were continuously digitized during the experiment by the data input system at a sampling rate of 5 kHz. The study was approved by the Ethics Committee at Fukuoka Women’s University and subjects gave prior written, informed consent. Appropriate compensation was given to the subjects after the experiment.

**Table 1:** Combinations of light stimuli

	**Masking-cone stimuli**	**Test stimuli**
1	None	mRGC
2	None	Cone
3	Present	mRGC
4	Present	Cone

The four combinations of light stimuli were randomly presented.

### Preliminary result with light flux stimulus

**Figure 3:**
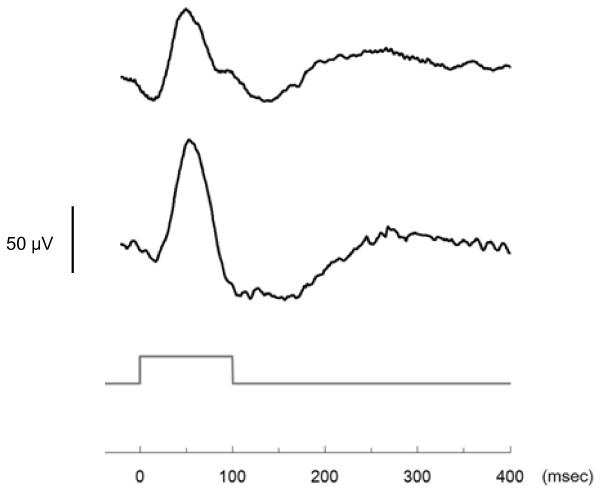
Preliminary results showing the ERG with light flux stimulus.

In order to assess validity of the ERG measurements, preliminary data from two subjects (females, 22 years old) were obtained with a light flux stimulus (often used in conventional ERG measurements). The light flux, with a maximum luminance of 1,132 cd/m^2^ for 100 msec, was given once to each subject after a 30-minute dark adaptation. The waveforms of the a- and b-waves were consistent with typical dark-adapted ERG characteristics with regard to implicit times: a-wave, about 20 msec; b-wave, about 50 msec (Figure3). The amplitudes of the a-wave and b-wave were approximately −15 μV and 50 to 80 μV, respectively.

### Analysis method

The ERG data were statistically analyzed in order to compare objectively cone and mRGC responses and verify effects of the masking-cone stimuli. Before statistical analyses, a 100-point moving-average was used to reduce noise from waveforms. The averaged ERG voltages before each test stimulus (over 20 msec) were regarded as baseline. Maximum positive deflections from the baseline were automatically detected at between 70 and 90 msec for the first peak and between 270 and 290 msec for the second, and maximum negative deflections were at between 150 and 170 msec for the first trough and between 340 and 360 msec for the second. The detection ranges were decided according to the average waveforms we observed. As the subject number (n = 5) was relatively small, data from all ERG responses to 30 test stimuli for each subject were used. The data were analyzed by SPSS (Ver. 20, IBM, Tokyo, Japan), and P < 0.05 was considered to be statistically significant. To analyze the time-course of the ERG responses, differences from baseline were averaged each 50 msec during the interval 0 to 1,000 msec after the stimulus, and were then analyzed by one-way ANOVA for repeated measurements.

## Results

**Figure 4:**
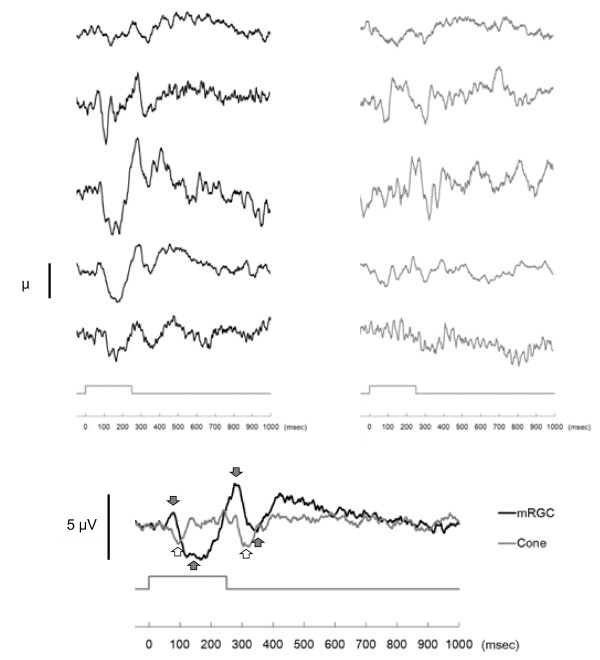
**Responses to the mRGC and cone stimuli when the masking-cone stimuli were not presented.** The averaged ERG responses of five subjects are shown following stimulation of the mRGCs (black line) and the cones (gray line). Upper panels show individual responses to the mRGC stimulus (left) and the cone stimulus (right), with adjacent panels coming from the same subject. The lower panel shows the averaged response. Black arrows show peaks and troughs in response to the mRGC stimulus and white arrows show troughs in response to the cone stimulus. The temporal envelope of the test stimulus is shown by the grey line above the time-scale axis.

**Figure 5:**
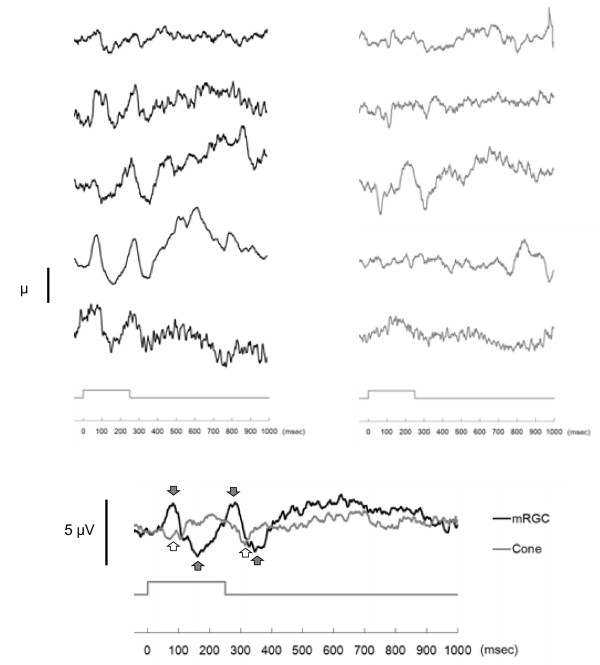
**Responses to the mRGC and cone stimuli when the masking-cone stimuli were given before the test stimulus.** The results are expressed in the same way as in Figure4.

Figure4 shows ERG responses of the five subjects to the mRGC stimulus (black line) and the cone stimulus (gray line) when the masking-cone stimuli were not presented. Figure5 shows ERG responses when the masking-cone stimuli were given before the test stimulus. In the case without the masking stimuli (Figure4), the averaged response to the mRGC stimulus (black line) had two peaks and two troughs. It increased after the onset of the mRGC stimulus, reached the smaller peak at approximately 75 msec (implicit time: 75 ± 9.4 msec, amplitude: 1.2 ± 0.4 μV, mean ± SD) and a trough at 120 to 180 msec; there followed a larger peak approximately 30 msec after the offset of the stimulus at approximately 280 msec (282 ± 10.4 msec, 3.6 ± 2.3 μV) and a second trough approximately 100 msec after the offset of the stimulus at approximately 350 msec. By contrast, there were no such positive peaks but only two negative troughs when the cones were stimulated (gray line); the first trough at approximately 100 msec (96 ± 9.5 msec, -2.2 ± 1.3 μV), a period of electro-neutrality between 150 and 250 msec, and a second trough approximately 65 msec after the offset of the stimulus at approximately 315 msec (316 ± 10.3 msec, -2.6 ± 0.5 μV).

In the case with the masking stimuli (Figure5), the mRGC response (black line) showed two positive peaks at approximately 80 msec (79 ± 6.5 msec, 2.5 ± 1.6 μV) and at approximately 280 msec (279 ± 13.4 msec, 2.9 ± 11 1.8 μV), and two troughs at 110 to 180 msec and 320 to 370 msec. The cone response (gray line) showed two troughs at approximately 100 msec (98 ± 19.6 msec, -1.7 ± 1.5 μV) and at approximately 315 msec (314 ± 7.4 msec, -1.8 ± 1.1 μV). These results were similar to those without the masking-cone stimuli (Figure4). For both mRGC and cone responses, neither amplitudes nor implicit times showed significant differences between the cases with or without the masking-cone stimuli (three-way ANOVA). Significant interactions were found between masking/non-masking, mRGC/cone stimuli and subjects. There was no significant difference among implicit times in the subjects, indicating that ERG responses showed the same characteristics to a stimulus in the subjects.

Regarding cone responses in the cases with/without the masking stimuli, the time-courses of the responses did not show significant variation from the baseline (repeated measurement using one-way ANOVA), and there were no significant differences between the amplitudes of the troughs (Wilcoxon signed-rank test). Despite this, variances (standard deviations) of the responses during the first 400 msec (when the waveforms seemed to have been affected by stimuli and peaks appeared) after the stimulus in the masking-cone condition were significantly smaller than those in the non-masking condition (Levene test, *P* < 0.001). Since the change in variation could be associated with the effect of the masking-cone stimuli in the cone-mediated pathway, the masking stimuli caused smaller amplitude deflections from the baseline in the ERG.

In the case with the masking stimuli (Figure5), the ERG response to the mRGC stimulus showed a significant change in time-course (one-way ANOVA with repeated measures, *P* < 0.01), while no significant differences from baseline were observed with the response to the cone stimulus. There was no clear indication that the responses to the mRGC and cone stimuli during 400 to 1,000 msec post-stimulation were different (two-way ANOVA). Since there were positive peaks at approximately 80 msec and approximately 280 msec in the response to the mRGC stimulus, maximum positive peaks from baseline at approximately 80 msec and approximately 280 msec were compared with those in the response to the cone stimulus. The response to the mRGC stimulus was significantly higher than that to the cone stimulus at approximately 80 msec (Wilcoxon signed-rank test, *P* < 0.05) and tended to be higher than the cone stimulus at approximately 280 msec (Wilcoxon signed-rank test, *P* = 0.08). These results show that ERG responses to the mRGC and cone stimuli had different characteristics.

## Discussion

We focused on describing features of waveforms rather than comparing peak amplitudes. When responses of the mRGCs and cones are compared, it is necessary to take into account their sensitivities. For instance, only the mRGC is sensitive enough to be able to respond to a single photon [27]. Responses of the mRGC are approximately 100 times larger than those of the cones [27], while the population of mRGCs is much smaller [8,28]. Therefore, in order to focus on distinguishing characteristics of the mRGCs and cones, the maximum stimulus contrasts for these two receptors (0.5 and 0.3, respectively; available in our illumination system) were adopted.

In spite of the masking-cone stimuli before the test stimulus, both the mRGC and cone responses showed similar characteristics to those in the non-masking condition. With regard to cone responses, the two troughs seem to correspond to the onset and offset, respectively, of the cone response (Figure5, gray line). Moreover, it was observed that the first trough was different from the a-wave of a standard cone response [29]. For example, the light intensity that initiates the a-wave is higher than that required to produce the b-wave, although a report has shown that there is a special case when only the a-wave appears - if knockout mice lacking metabotropic glutamate receptor subtype 6 in ON-bipolar cells are stimulated by light [30]. That is, the a-wave always accompanies the b-wave in conditions of ocular health. However, our data did not show any specific b-waves after the troughs. Also, the first trough, which was at approximately 100 msec in cone responses both with and without the masking-cone stimuli, was considerably later than the implicit time of the a-wave in the light-adapted ERG (10 to 20 msec is the standard value) [29]. Furthermore, the second trough, which is attributed to the offset of the stimulus, has not been reported in healthy subjects, and this aspect of the waveform appears to be unique to cone responses. These unique waveforms were obtained after stimulation of the cones by the illumination system, which indicates that the cone responses obtained by using normal ERG measurements show combined responses of cones and other cells, such as mRGCs and bipolar cells in the retina.

Although the b-wave is commonly accepted as being due to depolarization of ON-bipolar cells and evoked in response to the onset of a light flash [13,14], two positive peaks appeared in the mRGC response while no such peaks were present in the cone response (Figure5). One possible reason for no positive peaks in the cone response is that the stimulus to the cones was insufficient to induce a response after adaptation to the background stimulus. In this study, therefore, these two peaks appear, instead, to be responses of the mRGC to the onset and offset of the light stimulus. This result indicates that the mRGC depolarization contributes, at least partly, to both the b- and d-waves of the ERG. As for the implicit time of the first positive peak of the mRGC response (approximately 80 msec), it is longer than accepted values for the b-waves due to rod and cone responses (approximately 60 msec for the rods and approximately 30 msec for the cones) [29]. Although differences of experimental conditions between previous reports and ours should be considered, we stress that our illumination system stimulated mRGCs independent of rods and cones, and this observed delay indicates that the implicit time might reflect mRGC’s sluggish responses [5]. It is still possible that these peaks reflect combined responses of the mRGC and other cells (such as bipolar cells or amacrine cells), induced by the rods if the irradiance of the background stimulus is not high enough to saturate the rods. Although the irradiance of the background (534 cd/m^2^) is considered to be high enough in the conventional photopic ERG, it is necessary to investigate animal models to elucidate how mRGCs and other cells contribute to depolarization in the ERG. In addition, more indirect ways of studying mRGC responses in humans, such as action spectral sensitivity of melatonin suppression to light stimuli, could aid in understanding how much each photoreceptor contributes to circadian rhythm regulation.

Although additional data are required for a fuller interpretation of our results, this is the first report of amplitudes and implicit time in the ERG due to responses of the mRGC independent of rods and cones using the four-primary illumination system. The results show that the ERG responses to the mRGC and cone stimuli had different characteristics. That is, our illumination system successfully modulated stimulus levels to the mRGC and cones independently, and the differences in the responses could be measured in the ERG. It is suggested that our method will enable us to investigate separate behavior of cones and mRGCs in the circadian system, in collaboration with other approaches, such as studies of circadian rhythm behavior in animals and hormonal rhythms in humans.

## Conclusions

In the mRGC response, two positive peaks were observed approximately 80 and 280 msec after the beginning of the test stimulus and appeared to be responses to the onset and offset of the stimulus. By contrast, such peaks did not appear when the cones alone were stimulated. Although further work is required to interpret in more detail the ERG waveforms obtained in this study, it seems that these ERG waveforms show independent responses of the mRGCs. This is the first report of amplitudes and implicit time in the ERG from the responses of the mRGC that is independent of rods and cones and obtained using the four-primary illumination system. These results provide further knowledge about the mRGCs, which contribute to both visual and non-visual pathways, and increase understanding of visual science, neurophysiology and chronobiology.

## Abbreviations

mRGCs : melanopsin-expressing retinal ganglion cells; ERG : electroretinogram; LED : light-emitting diode.

## Competing interests

The authors have no competing interests.

## Authors’ contributions

YF designed and carried out the study, performed the statistical analysis and drafted the manuscript. SH and AY participated in the coordination of the study and helped to modify the manuscript. TM conceived of the study, and participated in its design and coordination and helped to draft the manuscript. All authors read and approved the final manuscript.
